# Angiogenesis, Neurogenesis and Neuroplasticity in Ischemic Stroke

**DOI:** 10.2174/157340310791658802

**Published:** 2010-08

**Authors:** M. Angels Font, Adriá Arboix, Jerzy Krupinski

**Affiliations:** 1 Fundació IDIBELL, Barcelona, Spain; 2 Department of Neurology, Hospital Universitari Sagrat Cor, Barcelona, Spain; 3 Cerebrovascular Diseases Unit, Department of Neurology, Hospital Universitari Mutua de Terrassa, Terrassa (Barcelona), Spain

**Keywords:** Angiogenesis-neurogenesis-stroke-plasticity.

## Abstract

Only very little is know about the neurovascular niche after cardioembolic stroke. Three processes implicated in neurorepair: angiogenesis, neurogenesis and synaptic plasticity, would be naturally produced in adult brains, but also could be stimulated through endogen neurorepair phenomena. Angiogenesis stimulation generates new vessels with the aim to increase collateral circulation. Neurogenesis is controlled by intrinsic genetic mechanisms and growth factors but also ambiental factors are important. The leading process of the migrating neural progenitor cells (NPCs) is closely associated with blood vessels, suggesting that this interaction provides directional guidance to the NPCs. These findings suggest that blood vessels play an important role as a scaffold for NPCs migration toward the damaged brain region. DNA microarray technology and blood genomic profiling in human stroke provided tools to investigate the expression of thousands of genes. Critical comparison of gene expression profiles after stroke in humans with those in animal models should lead to a better understanding of the pathophysiology of brain ischaemia. Probably the most important part of early recovery after stroke is limited capacity of penumbra/infarct neurones to recover. It became more clear in the last years, that penumbra is not just passively dying over time but it is also actively recovering. This initial plasticity in majority contributes towards later neurogenesis, angiogenesis and final recovery. Penumbra is a principal target in acute phase of stroke. Thus, the origin of newly formed vessels and the pathogenic role of neovascularization and neurogenesis are important unresolved issues in our understanding of the mechanisms after stroke. Biomaterials for promoting brain protection, repair and regeneration are new hot target. Recently developed biomaterials can enable and increase the target delivery of drugs or therapeutic proteins to the brain, allow cell or tissue transplants to be effectively delivered to the brain and help to rebuild damaged circuits. These new approaches are gaining clear importance because nanotechnology allows better control over material-cell interactions that induce specific developmental processes and cellular responses including differentiation, migration and outgrowth.

## 1. BASIC MECHANISMS IN CARDIOEMBOLIC STROKE AND NEUROREPAIR

Atrial fibrillation is the most common cardiac arrhythmias, and a major cause of morbidity and mortality due to cardioembolic stroke. The left atrial appendage is the major site of thrombus formation in non-valvular atrial fibrillation. Loss of atrial systole in atrial fibrillation and increased relative risk of associated stroke point strongly toward a role for stasis of blood in left atrial thrombosis. However, thrombus formation is multifactorial, and much more than blood flow irregularities are implicated. The impact of thrombus formation, its later migration towards the smaller vessels, subsequent stroke leads to very different cellular and vascular responses as compared to stroke due to atherosclerosis. Only very little is know about the neurovascular niche after cardioembolic stroke. Further, not much is known about the genetic basis for cardioembolic stroke. Only recently, an aging-suppressor gene, *klotho*, a candidate factor for vascular disease, its deficiency leads to impaired endothelium-dependent vasodilataion and imaipred angiogenesis, was demostrated as a genetic risk factor for ischemic stroke caused by cardioembolism in Korean females [1]. 

There are three mechanisms related with brain plasticity (anatomical and functional changes of the central nervous system with the aim to improve functional recovery)[2, 3]:

1. Brain circuits regulation with the activation of parallel pathways for restore impaired functions;
2. Unmasking of silent functional pathways.
  Both would be short term mechanisms
3. A third mechanism, typical for longterm plasticity, would be the production of new sprouts and dendritic spines in survival neurons as well as formation of new synapses.

Therefore the three processes implicated in neurorepair (angiogenesis, neurogenesis and synaptic plasticity) would be naturally produced in adult brains and after different pathological situations, but also could be stimulated through endogen neurorepair phenomena with different pharmacological treatments, always having in mind that the therapeutical window for neurorepair drugs is more wide than for neuroprotective drugs, being able to treat almost all the stroke patients [4]. It is well known that angiogenesis stimulation generates new vessels with the aim to increase collateral circulation. Angiogenesis is also directly related with neurogenesis since blood supply is necessary for new neuronal survival and development [2, 3].

Studying mechanisms regulating above processes will help to design future strategies in neurorepair. 

## 2. ANGIOGENESIS

### Nature Promotes Angiogenesis

After stroke, increased vascular remodelling is found in the areas of newly-born neuroblasts which migrate from the subventricular zone to the peri-infarcted cortex [5]. The presence of microvascular endothelial cells is important as they secrete growth factors and chemokines, which may support the survival of newly formed neurones. The administration of human cord blood-derived CD34+ cells following stroke induces neovascularisation in the peri-infarcted cortex and increases neuroblast migration to the damaged tissue [6]. However, the role of the angiogenesis after stroke may be dual. Collateral revascularization may be important in determining patient recovery from stroke. Indeed, higher microvessel density in the penumbra areas correlated with longer times of survival [7]. In order to be of benefit, increased microvessel density should persists in infarcted areas of the brain. In animal models, the long-term stability of ischaemia induced microvessels after middle cerebral artery occlusion (MCAO) show them leaky and transient. This newly formed blood vessels may serve to remove necrotic tissue and promote angiogenesis.

### Pathophysiology of Angiogenesis after Stroke

The permanent or transient middle cerebral artery occlusion models (pMCAO and tMCAO) are the major animal models for studying changes in gene or protein expression after stroke [8]. In the tMCAO model, ischaemia is generated for a fixed, transient time and reperfusion is then allowed. The changes which occur, most usefully mimick situation which occur after stroke in humans. However, limited knowledge about the molecular mechanisms involved in tissue regeneration has been gained from animal experiments using the MCAO model which replicates, in many aspects, the neuropathological changes seen following stroke in humans [9]. In addition, only the ipsilateral side of the brain is affected by the ischemic damage, allowing collection of experimental and reference control tissue from the same animal.

Angiogenesis and neurogenesis has been observed in the brains of patients who survived form several days to weeks after cerebral stroke and a positive correlation between microvessel density and patient survival was demonstrated [7]. Increased synthesis of angiogenic growth factors like FGF-2, PDGF, VEGF and its receptors was seen in the brain after stroke [7, 10, 11]. The molecules were up-regulated within hours of stroke and correlated with blood vessel growth in the penumbra region. The highest expression of molecules such as hyaluronidases, which degrade hyaluronan acid (HA) to an angiogenic low molecular weight form and its receptors on endothelial cells was seen in the acute phase of stroke, when remodelling may be occurring [12]. Analysis of ischemic brain tissue with techniques which are capable of studying multiple transcripts simultaneously can identify gene expression changes previously not known to be implicated in ischemic pathophysiology, and evaluation of their physiological significance. This can lead to development of new targets for stroke therapy [13]. DNA microarray technology has provided tools to investigate the expression of thousands of genes in a single hybridization experiment. Several experimental studies have examined alteration of gene expression in the post-ischemic rat brain, using microarray technology [14-18] while promising results of blood genomic profiling in human stroke have been obtained in recent pilot studies [19, 20]. Until recently, gene expression profiling had not been applied to patients dying of ischaemic stroke, in part because human brain autopsies are not regularly obtained. The majority of RNA transcripts and proteins in the human brain degrade only to a minor degree following death, thus making autopsy tissue a useful source for the isolation of nucleic acids and proteins [21]. In fact, previous studies evaluating the mRNA quality in human post-mortem brain tissue have demonstrated a minimal effect upon their overall relative stability within 24 hours of death [22-24]. Recently, Vikman and Edvinsson [25] investigated the gene expression in human middle cerebral artery (MCA) after ischaemia using human MCA samples 7-10 days post-stroke; however, they obtained their samples after a considerable delay of 2-3 days post-mortem and they focused mainly on mRNA expression of receptors. Critical comparison of gene expression profiles after stroke in humans with those in animal models should lead to a better understanding of the pathophysiology of brain ischaemia and will allow us to evaluate the usefulness of animal models in stroke research.

Pro-angiogenic factors have been described, being some of them the proinflammatory cytokines (IL-1beta, TNF-alfa), nitric oxide (NO), and growth factors (TGB-beta, PDGF, VEGT, FGF) that are also expressed after a ischemic process [2, 3, 26]. VEGF administration and VEGFR-1 modulation stimulates both neurogenesis and angiogenesis after a ischemic disease [27]. Recent studies in neonatal rodent stroke models suggest that recovery is due in part to upregulation of hypoxia-inducible factor-1-a and its downstream target, vascular endothelial growth factor. Vascular endothelial growth factor is upregulated after a hypoxic insult and is involved in neuronal survival, angiogenesis, and neurogenesis during the recovery process [28].

Angiogenesis is directly linked to neurogenesis. The later needs new vasculature in order to survive for a longer time. Mechansisms of angiogenesis are similar to neurogenesis and both processes share common factors. All three processes i.e. plasticity, neurogenesis and angiogenesis occur in adult brain as a response to injury but can be stimulated by additional cellular therapy. 

## 3. NEUROREPAIR: ANGIOGENESIS NEUROGENESIS AND PLASTICITY

### Triggering Penumbra

Ischaemic stroke results from cessation of blood flow in a major cerebral vessel and leads to de-regulation of genes whose expression promotes ischemic neuronal death and subsequent neurological dysfunction [29, 30]. Under ischemic conditions, due to the severe shortage of blood flow, energy metabolism fails, and severe reduction in mRNA and protein synthesis occurs in the ischemic core region. The tissue surrounding this area (peri-infarcted region) is able to maintain some functions such as ionic homeostasis and can be partially salvaged by blood recirculation (for a review see [2, 31]). The precise molecular mechanisms involved in ischemia-induced brain injury remain poorly understood.

The original definition of the ischaemic penumbra referred to areas of the brain that were damage but not yet dead, offering the promise that if proper therapies could be found, one could rescue brain tissue after stroke. Probably the most important part of early recovery after stroke is limited capacity of penumbra/infarct neurones to recover. It became more clear in the last years, that penumbra is not just passively dying over time. It is also actively recovering. In penumbra, partially depolarised tissue adjacent to the infarcted core with reduced cerebral blood flow and increased oxygen extraction rate struggle for survival in a very limited time-window. This initial plasticity in majority contributes towards later neurogenesis, angiogenesis and final recovery. Penumbra is a principal target in acute phase of stroke. Understanding of each of the processes is a major challenge for future therapies in neuroreparation. 

Many common pathways are triggered after stroke [32]. Possibly, the most important discovery is the biphasic nature of molecular signals in the penumbra in terms of injury versus repair [33]. This is the case for NMDA receptors or metalloproteinase (MMP) family. In the early phase after stroke these pathways may be detrimental, but in the contrary, in later phase are crucial for neuroblast migration and neurorepair [34-36]. 

### Neurogenesis

Neurogenesis, discovered only recently continues after the birth [37] and can be reactivated as a response to lesion [38]. Generation of new neurones from progenitors occurs in selected areas: subgranular zone of dentate gyrus, subventricular zone of some cortical areas, substantia nigra and perinfarcted areas. Neurogenesis is controlled by intrinsic genetic mechanisms and growth factors but also ambiental factors are important like physical activity, etc. [39]. The leading process of the migrating neural progenitor cells (NPCs) is closely associated with blood vessels, suggesting that this interaction provides directional guidance to the NPCs. These findings suggest that blood vessels play an important role as a scaffold for NPCs migration toward the damaged brain region [40]. Close impact on neurogenesis is contribution of hypoxia-inducible factor-1alpha (HIF-1alpha) to the therapeutic effect of neural stem cell (NSC) transplantation in cerebral ischemia. The relative efficacy of modified NSC to promote behavioral recovery was investigated in a rat model of stroke induced by a transient middle cerebral artery occlusion (MCAO). All animals showed functional improvement. Improvement was accelerated in animals receiving either NSC-Ad or Ad-HIF-1alpha, while improvement at all times between 7 days and 28 days post MCAO was significantly greater in animals transplanted with NSC-Ad-HIF-1alpha than for other treated animals. NSC-Ad-HIF-1alpha cells also increased the number of factor VIII-positive cells in the region of ischemic injury, indicating that HIF-1alpha expression can promote angiogenesis. Gene-modified NSC expressing HIF-1alpha have therapeutic potential in ischemic stroke [41-43]. Neural stem cells (NSCs) persist in the forebrain subventricular zone (SVZ) within a niche containing endothelial cells. Evidence suggests that endothelial cells stimulate NSC expansion and neurogenesis. Experimental stroke increases neurogenesis and angiogenesis, but how endothelial cells influence stroke-induced neurogenesis is unknown. SVZ neurospheres co-cultured with endothelial cells generated more immature-appearing neurons and oligodendrocytes, and astrocytes with radial glial-like/reactive morphology than controls. Oxygen glucose deprived endothelial cells stimulated neuroblast migration and yielded neurons with longer processes and more branching. These data indicate that intact and injured endothelial cells exert differing effects on NSCs, and suggest targets for stimulating regeneration after brain insults [44].

### Plasticity

Plasticity is defined as anatomic and functional changes in the central nervous system (CNS) which result in better functional recovery [45, 46]. The mechanisms involved are: regulation of brain circuits and activation of parallel pathways in order to maintain lesioned function; activation of silent pathways; and synaptogenesis leading to formation of new connections [47].

Acute brain lesions trigger diaschisis phenomena [48]. The diaschisis produces sudden loss of functions that are in areas remote to the injury but with anatomical connections to the damaged area. One of the mechanisms responsible for this depression of neuronal activity is glutamate excitotoxicity. The resolution of diaschisis contributes to later functional recovery, and in that recovery is not only involved the ipsilateral hemisphere but also the contralateral, the cerebellum and the spinal cord. The time profile of the changes reflect different mechanisms. The unmasking of silent synapses can strengthen existing neural circuits by modulating GABAergic mechanisms through rapid de-bottlenecking of the existing GABAergic inhibition. The long-term changes are based not only in unmasking the silent synapses but in axonal regeneration with creation and changes in shape, number and type of synapses [47]. Dendritic spines are major postsynaptic targets of glutamatergic transmission in the adult brain and are subject to constant remodeling through the action of neurotransmitters, neurotrophic factors, newly synthesized synaptic proteins and gene expression [49]. Further, that one of the main determinants of synaptogenesis is training [50, 51].

## 4. ATHEROSCLEROSIS-CARDIOEMBOLISM-ANGIOGENESIS

Formation of unstable plaques is a key mechanism leading to atherothrombosis, the major cause for ischaemic stroke, myocardial infarction and peripheral arterial disease. Patients who have symptomatic thrombosis in one vascular bed are at increased risk of disease in other beds. However, the development of the disease in carotid, coronary and peripheral arteries may have different pathophysiology suggesting that more complex treatment protocols may have to be designed to reduce plaque development at different locations. Important developmental features of coronary and carotid plaque development and genetic differences are seen which contribute to plaque development as well as in the deregulation of gene and protein expression and cellular signal transduction activity of active cells in regions susceptible to thrombosis. Differences between carotid and coronary artery plaque development might help to explain the nature of embolic mechanisms of stroke [52].

Clinical signs of atherosclerosis, such as heart attack and stroke, are often caused by rupture of the cap of an atherosclerotic plaque, with thrombus formation as a consequence. The risk of rupture depends on the formation of microvessels (angiogenesis) in the plaque. The fragility of the microvascular endothelium causes hyperpermeability, which leads to intraplaque haemorrhage. Angiogenesis is stimulated by hypoxia, oxidative stress and the production of hypoxia-inducible transcription factors. Hypoxia is primarily caused by increased oxygen consumption of inflammatory cells, while plaque thickness, which reduces oxygen diffusion, contributes to a limited extent. A vicious circle of hypoxia, deletorious angiogenesis and inflammation occurs deep in the plaque, which enhances plaque growth and the risk of plaque rupture. By non-invasive imaging of plaque hypoxia and angiogenesis, plaques at risk of rupture may be identified. Therapeutic interventions for plaque angiogenesis and hypoxia require further investigation [53].

## 5. MODELING THE NEUROVASCULAR NICHE

Therapeutic strategies in stroke have been developed with two main aims: restoration of cerebral flow and the minimization of the deleterious effects of ischemia on neurons. Intense research spanning over the last two decades has witnessed significant therapeutic advances in the form of carotid endarterectomy, thrombolytics, mechanical thrombectomy, anticoagulant therapy, antiplatelet agents, neuroprotective agents, and treating associated risk factors such as hypertension, diabetes and hyperlipidemia. However, the search for an effective neuroprotectant remains frustrating, and the current therapeutic protocols remain suboptimal. Till date only one FDA-approved drug is available for ischemic stroke; i.e., the serine protease tissue-type plasminogen activator (tPA), utility of which is limited by short therapeutic window [54].

### Cellular Therapy

The main aim of cell therapy is to mimic neuroreparation processes that naturally occur in the brain.  At experimental level in animal models of cerebral ischemia, three types of human cells have been used for transplants: neural progenitor cells derived from fetal tissue, neural cell lines, stromal cells and hematopoietic progenitor/endothelial derived from bone marrow, umbilical cord blood, peripheral blood or adipose tissue [55]. Recent data suggest that bone–marrow stromal cells from stroke rats (Isch-BMSCs) are superior to normal rats (Nor-BMSCs) for the neurorestorative treatment of stroke, which may be mediated by the enhanced trophic factor and angiogenic characteristics of Isch-BMSCs [56].Currently there are few completed clinical trials in patients with ischemic stroke. The treatment has been shown safe and without side effects although functional improvement of the treated group compared to control is unclear [57]. The reality is that there are still many issues to resolve before cell therapy becomes a therapeutic option to treat stroke.

### Old or New Pharmacology

In our clinical practice, we use two basic methods of treatment, preventive treatment based on long-term use of antiplatelet or anticoagulant to reduce the risk of a new event, or fibrinolytic therapy as an acute treatment of stroke. However, less than 2% of patients receive the latter treatment. In last years researchers are working hard in search of neuroprotective agents in the acute phase of stroke and in the search for neurorepair drugs to use in the chronic phase. While the therapeutic window of neuroprotective drugs is small (0-8 hours at best), the window of neruorepair is much wider (days, weeks or even months).

Drug therapy is oriented towards stimulating endogenous neurorepair processes. In the absence of succesful drug therapy, rehabilitation is the most useful treatment for improving functional recovery after stroke [51, 58]. As a general rule, most neurorepair drug trials generally showed that drugs are safe, but the number of patients included is small and there is no contrasting results that allow recommendation for larger population [58-60]. Drugs affecting noradrenergic transmission such as amphetamine, methylphenidate or levodopa have been tested, or drugs that act at nitrergic level, as donors of NO, sildenafil or analogues of cyclic GMP (disputed by the dual role of NO in neurogenesis). Other drugs that have been tested are selective inhibitors of serotonin reuptake, myelin inhibitors such as anti-Nogo-A (myelin prevents axonal growth and limits synaptic plasticity), botulinum toxin, erythropoietin or the hopeful statins and citicoline.

More recent studies demonstrated beneficial effects of G-CSF treatment (granulocyte colony-stimulating factor) in various CNS disease. Possible mechanisms underlying this activity are neuroprotection, anti-apoptosis, angiogenesis and anti-inflammation. In combination with G-CSF-induced leukocytosis, increased peripheral neutrophils could aggregate within microvasculature and additionally impair blood perfusion of the ischemic tissue. Authors demonstrated that G-CSF deficiency leads to increased infarct volumes, whereas G-CSF substitution revokes detrimental effects by reducing lesion size and enhancing neurological outcome compared to untreated animals. Administration of G-CSF is accompanied by significant increase of circulating neutrophils 2 days post-ischemia but leukocytosis is restricted to the vessel compartment and has no deleterious effect on lesion formation and functional recovery. These observations are likely to be important for therapeutic targeting of G-CSF-mediated neuroprotection in stroke [61, 62]. Another new possible neuroprotective target is activated protein C (APC). In stroke models, promotes postischemic neovascularization and neurogenesis. Mice treated with single-dose or multidose APC, compared with vehicle, showed significantly improved motor function on all tests. In the single-dose and multidose APC treatment groups, at 7 days after treatment, lesion volume was significantly decreased by 30% and 50%, respectively. Multidose APC, but not single-dose APC,  days after treatment, lesion volume was significantly decreased by 30% and 50%, respectively. Multidose APC, but not single-dose APC, increased new blood vessel formation. Multidose APC also promoted proliferation of neuroblasts in the subventricular zone (SVZ) and their migration from the SVZ to the perilesional area. In conclusion activated protein C improves functional outcome and is neuroprotective. It also promotes angiogenesis and survival and migration of neuroblasts from the SVZ to the perilesional area, but the exact role of these brain repair mechanisms remains to be determined [63].

### Therapeuctic Potential of Angiogenesis

The origin of newly formed vessels and the pathogenic role of neovascularization and neurogenesis are important unresolved issues in our understanding of the mechanisms after stroke. Furthermore, the lack of consensus concerning the contribution of angiogenesis has serious practical implications because it continues to place a question mark on the use of angioneurins to treat stroke. Clearly, more experimental work is needed to address these questions and illuminate some of the key outstanding problems. Among the currently available treatments, only statins, showed pleiotropic pro- and antiangiogenic properties. The currently available data from both animal and human studies regarding the effects of statins on angiogenesis demonstrate that statins are safe, orally available agents that may acquire novel therapeutic indications through their angiogenic modulating effects [64]. Combination of sub-therapeutic doses of Simvastatin and bone marrow stromal cells (BMSCs) have additive effects in stroke therapy, improves neurological outcome, enhances angiogenesis and arteriogenesis, and increases the number of engrafted-BMSCs in the ischemic brain. Simvastatin significantly increased stromal cell-derived factor-1 (SDF1) expression in the ischemic brain and chemokine (CXC motif) receptor-4 (CXCR4) in BMSCs, and increased BMSC migration to RBMECs and astrocytes. Thus, combination treatment of stroke upregulates the SDF1/CXCR4 axis and enhances BMSC migration into the ischemic brain, amplifies arteriogenesis and angiogenesis, and improves functional outcome after stroke [65].

Controlled reperfusion and re-establishment of the local micro-circulation, together with reduction in both immediate and delayed neuronal apoptosis, after stroke, could improve neuronal survival and organization, and ultimately patient recovery [66]. Cell-based angiogenic therapy after cerebral ischaemia not only induces angiogenesis but improves neuronal regeneration [6]. Investigation into the factors that promote angiogenesis (the growth of new blood vessels from pre-existing ones) and neuroprotection, which over a period of at least a few days might help limit neuronal injury, might identify a target for therapeutic intervention in stroke. 

In ischaemic penumbra, normal cell function might be retained by restoration of the blood flow to such areas, and hence the penumbra is salvageable by therapeutic intervention [2]. Neurotrophic factors, for instance nerve growth factor (NGF), brain-derived neurotrophic factor (BDNF) and basic fibroblast growth factor (FGF- 2) are involved in the regulation of nerve cell survival and differentiation during development and in the functional maintenance of adult neurones [67, 68]. FGF-2 is also thought to be important for regeneration and restoration of function in pathophysiological situations, such as chemical neurotoxicity, mechanical trauma and brain ischaemia and more specifically can protect cortical synaptic terminals from amyloid and oxidative stress-induced impairment of glucose, glutamate transport and mitochondrial function. The major progress in neurovascular signalling was discovery that many molecules affect the development of both the nervous system and the vascular system [69]. Some classical angiogenic factors described above, have originated from the nervous system and some of the classical neurotrophic factors have also angiogenic properties. These molecules that have such dual neurovascular properties are currently called angioneurins and are divided in three major groups: (1) angioneurins discovered through their neuronal effects (NGF, BDNF, NT3, NT4), (2) angioneurins discovered as angiogenic factors (VEGF, PDGF, ANG), (3) other angioneurins that have pleiotrophic effects (TGF, EPO, FGF2, HGF, EGF, IGF1, PGRN) [69]. An important property of angioneurins is their neuroprotective activity. They enhance neurogenesis, modulate synaptic plasticity, have positive effect on neurite outgrowth, branching and elimination making them ideal neuroprotective Drugs Madri JA *et al*., [70] using *in vivo* and *in vitro* murine models of sublethal hypoxia mimicked the variable responses observed in the human population and correlated differences in baseline and hypoxia-induced induction of HIF-1alpha and several downstream signaling components including BDNF, VEGF, SDF-1, TrkB, Nrp-1, CXCR4 and NO with differences in survival as well as endothelial cell and neural stem cell survival and proliferation, providing insight into this important and timely problem and suggesting that optimization of expression levels of some or all of these signaling components may have the potential of maximizing recovery following CNS injury [71]. New question raised recently is whether patients with different risk factors may have variable responses to proangiogenic therapies. Indeed, the development of collateral vessels, which is important to prevent ischemic tissues from cell death, is impaired in patients with diabetes mellitus. The process is regulated by many positive and negative factors. Compared with the controls, the diabetes groups have lower vessel density, more expression of angiostatin, and lower level of VEGF. These results showed angiogenesis is deficient in diabetes groups after ischemical reperfusion (I/R) injury. And the possible mechanism is hyperglycemia attenuates neovascularization by downregulating proliferative properties of VEGF and upregulating of negative properties of angiostatin [72]. Novel emerging target is beta1 integrin, a cell surface molecule that is critical for endothelial cell adhesion, migration and survival during angiogenesis. beta1 integrin plays important roles in neurovascular remodelling and functional outcomes following stroke, and that targeting the beta1 integrin signalling may provide a novel strategy for modulating angiogenesis in ischemic stroke and other pathological conditions [73].

### Biomaterials for Neurorepair

Biomaterials for promoting brain protection, repair and regeneration are new hot target [74]. Recently developed biomaterials can enable and increase the target delivery of drugs or therapeutic proteins to the brain, allow cell or tissue transplants to be effectively delivered to the brain and help to rebuild damaged circuits. Similarly, biomaterials are being used to promote regeneration and to repair damaged neuronal pathways in combination with stem cell therapies. These new approaches are gaining clear importance because nano-technology allows better control over material-cell interactions that induce specific developmental processes and cellular respones including differentiation, migration and outgrowth. 

## 6. CONCLUSIONS

Dissection of mechanisms and molecular pathways that mediate neuronal death should bring major advances in successful neuroprotective therapies. However, this purely neuronal approach failed in the past decades [31]. Neurones, as the most important cells in the CNS are integrated in a complex network of multiple cell types, including neurones, astrocytes, oilgodendrocytes, microglia, endothelial cells and pericytes comprising the cerebral microvasculature as well as matrix with axonal compartments in white matter [75]. The NINDS stroke progress review group identified the “*neurovascular unit*” as a conceptual model that emphasizes cell-cell and cell-matrix signaling between all the cells of the brain (NINDS, 2002). Further, integration between neuronal and vascular components considers the brain from the functional perspective. This is important for future research with neurovascular unit as a therapeutical target.

The major challanges for future lie in the uncoupling of biphasic response to ischaemia and dual effects of many molecules which are implicated in the initial tissue damage. Advances in molecular biology, genetic engineering, proteomics and genomics are increasing our understanding of disease processes and may soon allow us to treat or reverse the underlying pathology. At the same time, advances in cell-based therapeutics, regenerative medicine and tissue engineering are raising the possibility of replacing damaged neurons or coaxing neuronal circuits to regenerate. Here, biomaterials (i.e. materials that are used and adapted for a medical application and thus intended to interact with as biological system) have become increasingly important in the development of drug delivery systems and tissue engineering approaches. In the near future, they may play a key role in overcoming the inherent insufficiency of protection, repair and regeneration of the brain.
